# Antimicrobial Resistance in Commensal *Escherichia coli* of the Porcine Gastrointestinal Tract

**DOI:** 10.3390/antibiotics12111616

**Published:** 2023-11-11

**Authors:** Lorcan O’Neill, Edgar García Manzanilla, Daniel Ekhlas, Finola C. Leonard

**Affiliations:** 1 Pig Development Department, Teagasc, The Irish Food and Agriculture Authority, Moorepark, Fermoy, Co Cork P61 C996, Ireland; edgar.garciamanzanilla@teagasc.ie (E.G.M.); daniel.ekhlas@ucdconnect.ie (D.E.); 2 School of Veterinary Medicine, University College Dublin, Belfield, Dublin D04 V1W8, Ireland; nola.leonard@ucd.ie; 3 Food Safety Department, Teagasc Food Research Centre, Ashtown, Dublin D15 DY05, Ireland

**Keywords:** antimicrobial resistance, *Escherichia coli*, commensal, pigs

## Abstract

Antimicrobial resistance (AMR) in *Escherichia coli* of animal origin presents a threat to human health. Although animals are not the primary source of human infections, humans may be exposed to AMR *E. coli* of animal origin and their AMR genes through the food chain, direct contact with animals, and via the environment. For this reason, AMR in *E. coli* from food producing animals is included in most national and international AMR monitoring programmes and is the subject of a large body of research. As pig farming is one of the largest livestock sectors and the one with the highest antimicrobial use, there is considerable interest in the epidemiology of AMR in *E. coli* of porcine origin. This literature review presents an overview and appraisal of current knowledge of AMR in commensal *E. coli* of the porcine gastrointestinal tract with a focus on its evolution during the pig lifecycle and the relationship with antimicrobial use. It also presents an overview of the epidemiology of resistance to extended spectrum cephalosporins, fluoroquinolones, and colistin in pig production. The review highlights the widespread nature of AMR in the porcine commensal *E. coli* population, especially to the most-used classes in pig farming and discusses the complex interplay between age and antimicrobial use during the pig lifecycle.

## 1. Introduction

*Escherichia coli* is the predominant aerobic microorganism in the microbiota of the vertebrate gastrointestinal tract [1,2]. Although primarily a commensal microorganism, several pathogenic strains are known to cause disease in both humans and animals [3]. Diarrhoeagenic strains of *E. coli* are an important cause of gastroenteritis in humans [4]. Some of these strains are zoonotic, with Shiga toxin-producing *E. coli* (STEC) representing the fourth most common bacterial food-borne infection in Europe, after campylobacteriosis, salmonellosis, and yersiniosis [5]. In humans, extraintestinal pathogenic *E. coli* (ExPEC) are the microorganisms most frequently implicated in urinary tract infections worldwide [6], and are the leading cause of bloodstream infections in Europe [7]. Antimicrobials are generally not indicated for the treatment of gastroenteritis caused by *E. coli* [8]. In contrast, they are essential in the treatment of ExPEC infections and, hence, antimicrobial resistance (AMR) in these bacteria is a major public health issue. Beta-lactams, cephalosporins (third generation and higher), fluoroquinolones, aminoglycosides, and carbapenems, all classified as critically important antimicrobials (CIA) by the World Health Organization (WHO) [9], represent the most important treatment options, and resistance to these classes in human isolates is monitored by the European Antimicrobial Resistance Surveillance Network (EARS-Net) [7]. This threat to public health is further highlighted by WHO’s inclusion of carbapenem and third-generation cephalosporin-resistant *Enterobacteriaceae* among its “Priority 1: critical” AMR pathogens for the development of new antibiotics [10].

**Figure 1:**
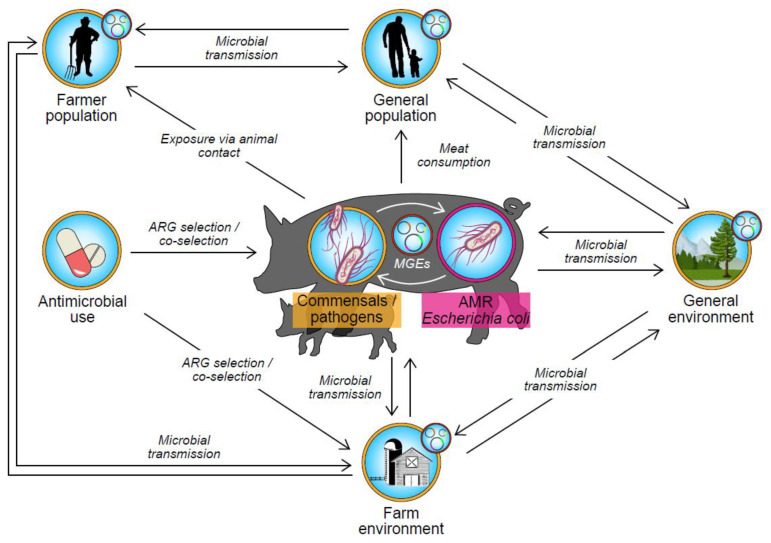
Schematic representation of potential transmission pathways of AMR *Escherichia coli* and/or their associated ARGs between pigs, humans, and the environment. The smaller circular icon associated with the human and environment ecosystems represents AMR transmission within the microbiota of the relevant ecosystem. Legend: AMR—antimicrobial resistance; ARG—antimicrobial resistance gene; MGE—mobile genetic element.

Antimicrobial resistance in *E. coli* of animal origin poses a threat to human health in two ways. Firstly, similarities between certain ExPEC strains and avian pathogenic *E. coli* (APEC) strains in poultry have led some authors to suspect that some ExPEC infections may be zoonotic [11,12,13,14] and, in particular, associated with the consumption of chicken [15,16]. Secondly, as illustrated in Figure 1, animals represent a reservoir of AMR *E. coli* to which humans may be exposed via the food chain, direct contact with animals, or environmental contamination [17]. These AMR *E. coli* may transfer antimicrobial resistance genes (ARG) to bacteria of human importance by various means of horizontal gene transfer (HGT). Such HGT events may occur in the animal host, for example, between *E. coli* and *Salmonella* spp. [18,19], with subsequent zoonotic transmission; within the human host, after transient colonisation by *E. coli* of animal origin and subsequent HGT to human commensal or pathogenic bacteria [20]; or in the external environment. Thus, the commensal *E. coli* population is considered to be an indicator for AMR in the wider Gram-negative bacterial population. Pig farming is one of the largest livestock sectors worldwide [21] and is the highest consumer of veterinary antimicrobials [22]. Therefore, the transmission of AMR *E. coli* of porcine origin to humans via the food chain, occupational exposure, or environmental contamination poses a threat to public health. While recent whole genome sequencing (WGS) studies suggest that animals do not contribute significantly to the overall burdens of either ExPEC infections [23] or AMR *E. coli* in humans [24,25,26], the risks cannot be discounted entirely. The potential exists for new pathogenic strains to emerge, which would be especially concerning if accompanied by AMR. Moreover, occupational exposure to pigs has been associated with increased carriage of AMR *E. coli* on Canadian farms [27], while in the Netherlands, closely related extended spectrum beta lactamase (ESBL) producing strains of *E. coli* and/or ESBL genes were identified in farm and abattoir workers and in the pigs to which they were exposed [28,29,30]. Therefore, a thorough understanding of the dynamics of AMR *E. coli* in the pig lifecycle is required to help mitigate current and future threats to public health. 

It should be noted that AMR in porcine bacteria is not limited to *E. coli*. Indeed, AMR is reported in a range of commensal and pathogenic bacteria, as can be seen in the reports of various national and international surveillance programmes [31,32,33], which include *Salmonella* spp., *Enterococcus* spp., *Campylobacter jejuni*, *Staphylococcus aureus*, *Actinobacillus pleuropneumoniae,* and *Brachyspira hyodysenteriae,* among others. Nor is AMR in porcine *E. coli* limited to commensal strains. Enterotoxigenic *E. coli* (ETEC) strains are implicated in neonatal and post-weaning diarrhoea in piglets, and treatment is frequently complicated by the presence of AMR [34,35]. However, this review focuses on AMR in commensal *E. coli* isolated from the gastrointestinal tract of healthy pigs. Published reviews on this topic include a systematic review analysing the relationship between AMR in *E. coli* and oral exposure to antimicrobials [36]; two recent systematic reviews examining resistance to extended spectrum cephalosporins, carbapenems, fluoroquinolones, and colistin [37,38]; and a review of AMR *E. coli* in China [39]. This literature review presents an overview and appraisal of current knowledge of AMR in commensal *E. coli* isolated from the porcine gastrointestinal tract in pig farming more generally, with a particular focus on its evolution during the pig’s lifecycle and the relationship with antimicrobial use (AMU). A brief review of the epidemiology of resistance to extended spectrum cephalosporins, fluoroquinolones, and colistin in pig production is also presented. 

Before presenting the review, it is important to consider the criteria used to interpret the antimicrobial susceptibility tests (AST), which determine the presence or absence of AMR. Different interpretive criteria can hamper direct comparison between studies, especially when quantitative data, i.e., minimum inhibitory concentrations (MIC) or zone diameters, are not available. Some of the studies presented here used clinical breakpoints (CBP) as their interpretive criteria. These are determined using MIC distribution, pharmacokinetic/pharmacodynamic and clinical outcome data, and thus describe ‘clinical resistance’ [40]. Other studies used epidemiologic cut off values (ECOFF) which divide the bacterial population according to the presence, i.e., non-wild type (nWT), or absence, i.e., wild type (WT), of acquired resistance mechanisms. These are determined using MIC or zone diameter data only and thus describe ‘microbiological resistance’ [41]. It is important to distinguish between microbiological and clinical resistance because the presence of an acquired resistance mechanism (whether determined phenotypically or genotypically) is not always associated with clinical resistance and, as such, ECOFFs should not be used as a basis for clinical decisions [41]. Notwithstanding the distinction between clinical and microbiological resistance, many authors use the terms ‘susceptible’ and ‘resistant’ to describe WT and nWT isolates, respectively (with or without explaining the distinction) and, although not strictly correct, do so in the interest of readability. Clinical breakpoints and ECOFFs can differ depending on whether European Committee on Antimicrobial Susceptibility Testing (EUCAST), Clinical and Laboratories Standards Institute (CLSI), or national guidelines (CBPs only) are used [40,42,43,44]. Furthermore, different CBPs may be defined for humans and animals (some studies used veterinary breakpoints) and both CBPs and ECOFFs may change over time (meaning that older studies may not be directly comparable to more recent ones). Finally, some studies may use other criteria, usually for the purposes of screening for specific resistance mechanisms, which are not always analogous to the relevant ECOFF or CBP (e.g., specific monitoring of ESBL/AmpC producing *E. coli* in food animals in Europe [33,45]). The various interpretive criteria discussed highlight a limitation in the field of clinical microbiology, generally, and in this review, specifically, regarding the comparison of results between studies. However, it would be impossible to discuss all of the disparities between the different studies during the narrative without hampering readability. Therefore, for the purposes of this review, the terms susceptible and resistant refer to the interpretive criteria used by the authors of the individual studies, although the reader should keep in mind the nuances of AST and its interpretation as they proceed through the text.

## 2. Antimicrobial Resistance in *Escherichia coli* of Porcine Origin: Surveillance Programmes

Antimicrobial resistance in *E. coli* of porcine origin is not a recent phenomenon. In 1957, Smith and Crabb reported that the continuous inclusion of tetracyclines in feed selected for tetracycline resistance in the commensal *E. coli* population in pigs [46], and a further longitudinal study conducted in the years after a ban on the use of medically important antimicrobials as feed additives/growth promoters in the United Kingdom (UK) showed that relatively high levels of tetracycline, streptomycin, and sulphonamide resistance had persisted and that trimethoprim resistance had emerged by the end of the 1970s [47]. Similarly, in Denmark, Albaek et al. (1991) showed that, despite similar restrictions on the use of growth promoters, the levels of resistance to tetracycline, streptomycin, and sulphonamide were significantly higher in 1988 than those reported in two earlier studies conducted in the 1970s [48]. Moreover, the prevalence of resistance to ampicillin, neomycin, and chloramphenicol, which were rare in the earliest of the comparison studies (3%, 0% and 3%, respectively), had also increased (84%, 47% and 30%, respectively) by 1988 [48]. More recently, commensal *E. coli* from food animals, including pigs, have been included with pathogenic bacteria in the AMR monitoring programmes of several countries, in line with recommendations by the WHO Advisory Group on Integrated Surveillance of Antimicrobial Resistance (AGISAR) [49]. Examples of these programmes outside of Europe include the National Antimicrobial Resistance Monitoring System (NARMS) in the USA [50], the Canadian Integrated Program for Antimicrobial Resistance Surveillance (CIPARS) [51], and the Japanese Veterinary Antimicrobial Resistance Monitoring System (JVARM) [52]. In Europe, the European Food Safety Authority (EFSA) and the European Centre for Disease Prevention Control (ECDC) oversee a programme that mandates European Union (EU) member states to monitor AMR in commensal *E. coli* from slaughter pigs biennially [33]. Some non-EU states also participate (e.g., European Economic Area (EEA) members, Switzerland) and, while the UK no longer participates since leaving the EU, it continues to publish its United Kingdom Veterinary Antibiotic Resistance and Sales Surveillance (UK-VARSS) reports using the same methodology [31]. Another pan-European programme, the European Antimicrobial Susceptibility Surveillance in Animals (EASSA), is operated by the pharmaceutical industry [53]. Several European states also operate their own monitoring programmes, for example, the Danish Integrated Antimicrobial Resistance Monitoring and Research Programme (DANMAP) in Denmark [54] and the Monitoring of Antimicrobial Resistance and Antibiotic Usage in Animals in the Netherlands (MARAN) programme in the Netherlands [55]. As these monitoring programmes mature, more detailed analyses, e.g., of multiyear trends, will become available, as is the case for Portugal, Denmark, and the USA [56,57,58]. Schrijver et al. (2018) reviewed the monitoring programmes in operation in Europe up until 2016 and found marked heterogeneity in sampling and laboratory methodology, as well as in the availability of results (i.e., language and frequency of publication) [59]. However, the EFSA and ECDC programme, with harmonised protocols and transparent reporting, allows for a comparison of AMR in the participating European countries. In contrast, monitoring programmes in lower- and middle-income countries (LMIC), including China, the largest pig producing country globally, are largely absent or at least not publicly available in the English language [60,61,62].

## 3. Resistance in *Escherichia coli* Isolated from Finisher Pigs: Data from Surveillance Programmes and Published Studies

The handling and consumption of pork is expected to represent the highest risk of human exposure to AMR *E. coli* of porcine origin in the general population. Therefore, national monitoring programmes and the majority of published studies sample pigs at or close to slaughter. Table 1 presents a summary of the most recent data from the EFSA and ECDC, UK-VARSS, NARMS, CIPARS, and JVARM programmes. These data show that resistance to tetracyclines, sulphonamides, trimethoprim, aminopenicillins, amphenicols, and aminoglycosides is common, reflecting the widespread use of these classes in pig production. Resistance to the highest priority critically important antimicrobials (HP CIA) [9] is generally lower, although high levels of fluoroquinolone resistance are observed in some individual European nations. It should be noted that, while streptomycin, an aminoglycoside, is not included in the EFSA and ECDC testing panel, relatively high levels of resistance are expected, in line with cross-sectional European studies [63,64,65,66,67]. High levels of streptomycin resistance, not evaluated in the most recent NARMS data but included previously [68], are also reported in the USA [58]. Table 2 summarises the results from a selection of studies that investigated AMR in commensal *E. coli* isolated from healthy finisher pigs on farm or at slaughter. Not intended to present an exhaustive list of all such studies carried out, comparisons between the studies included in this table should be made with caution. As discussed in the introduction, the criteria used to interpret the AST results can hamper direct comparison between studies. Differences in study design, for example sample size, the age of the animals at sampling, or the laboratory methods employed, may also impact the results obtained. Nevertheless, the results from the European and North American studies in Table 2 are broadly in line with those from their respective regional monitoring programmes, which, along with the results from other regions, confirm high prevalence of resistance to the heavily used antimicrobial classes in most settings, with some exceptions. In Europe, northern countries such as Sweden and Norway, with long histories of good antimicrobial stewardship, generally have lower AMR prevalence than southern European countries [33]. In Africa, AMR was generally lower in the less intensive production systems studied in Nigeria, Uganda, and Rwanda [69,70,71] than the intensive systems in Tanzania [72,73]. On the other hand, studies from LMICs in Asia suggest that AMR in intensive pig production is higher than in developed countries, particularly for HP CIAs. In China, the high rates of resistance to tetracyclines, sulfonamides, beta-lactams, amphenicols, and fluoroquinolones illustrated in Table 2 [74,75] are consistent with other studies in which the age and/or health status of the sampled pigs were uncertain [76,77,78,79,80,81] and with the findings of a recent meta-analysis [39]. They are also consistent with the estimated resistance rates determined in a pooled analysis of point prevalence studies that investigated AMR in *E. coli* isolated from pig farms, slaughterhouses, and food in China between 2000 and 2019 [62]. Interestingly, there is disagreement between some of the studies conducted in China concerning resistance to third-generation cephalosporins and to colistin. For example, colistin resistance was high in some studies, for example 46.3% [76] and 59.7% [82], but low or absent in others [74,77,80,81]. Such discrepancies could be due to differences in geographical area in such a large country, study design, or laboratory methods, and further highlight the benefits of systematic surveillance. In Southeast Asia, two studies in Thailand that investigated AMR in finisher pigs on groups of farms with different levels of AMU also reported high rates of resistance to most of the antimicrobials tested, especially on farms with routine prophylactic AMU, where almost all isolates were resistant to tetracycline, ampicillin, trimethoprim/sulfamethoxazole, and chloramphenicol [83,84]. Furthermore, rates of resistance to third-generation cephalosporins and fluoroquinolones in those studies were well in excess of those reported in developed countries. Similar results were reported in studies conducted at slaughterhouses in Vietnam, Cambodia, and Thailand [85,86], highlighting concerns that LMICs, especially in Asia, represent ‘hotspots’ of AMR [37,38,61].

## 4. Resistance in *Escherichia coli* Isolated from Pigs in Age Groups Other Than Finisher 

Much of the work investigating AMR in commensal *E. coli* in younger pigs involved experimental trials. These often involved using specific antimicrobial treatments [103] or were conducted on experimental farms or over limited timeframes [104], meaning that their findings may not be directly applicable to conditions in the field. Compared with the number of field studies investigating AMR in finisher pigs, relatively few have investigated AMR in commensal *E. coli* originating from other age groups. Many of these studies reported higher levels of AMR in younger animals, which is a phenomenon noted in various other species, including humans [105]. However, this varied somewhat between studies as temporal trends may differ depending on the antimicrobial and/or AMU patterns studied. Younger pigs, especially after weaning, are more likely to receive antimicrobials [106,107] which undoubtedly affects the prevalence of resistance. Studies in Ireland, Canada, USA, and Spain all showed higher rates of resistance in isolates from weaner pigs compared with finisher pigs for most, if not all, of the antimicrobials studied. [65,96,108,109]. Similarly, Pissetti et al. (2021) reported increased odds of a multidrug resistant (MDR) phenotype in isolates from nursery pigs compared with finisher pigs on Brazilian farms [110]. While the peak in AMR in *E. coli* during the weaner stage reported in these studies coincides with high AMU in this age group, the findings of other studies suggest that AMU alone may not explain this phenomenon. On a US research farm not exposed to antimicrobials for over five years, resistance to tetracycline, sulfisoxazole, and streptomycin was higher in isolates from weaner pigs compared with most of the older age groups [111]. More recently, Yun et al. (2021) reported that the prevalence of MDR on 10 Finnish farms was higher at 5 weeks of age compared with 22 weeks, regardless of whether they received antimicrobials during their lifetime or not [112]. A longitudinal study conducted on 29 farms in Germany tracked resistance to ampicillin, tetracycline, colistin, and azithromycin in pigs treated or not treated with the respective antimicrobial class [113]. Resistance to ampicillin and tetracycline in untreated pigs, as well as to azithromycin regardless of treatment status, peaked during the weaner stage. In contrast, the lowest levels of resistance were observed in finisher pigs with the exception of tetracycline resistance in tetracycline-treated pigs. In that study, the higher tetracycline resistance in treated finisher pigs was likely associated with tetracycline use in the later production stages, whereas the other antimicrobial classes were only used in younger pigs [113]. A cross-sectional study in the USA compared AMR in antibiotic free (ABF) and conventional herds (n = 3 and n = 4, respectively) [109] and found that the minimum inhibitory concentrations (MIC) of ampicillin, gentamicin, and sulfamethazine were highest in the two youngest age groups (pigs weighing 4.5 kg and 23 kg), but only on conventional farms. This was not the case for oxytetracycline, where resistance on the ABF farms was highest in the younger pigs, despite the absence of AMU, but highest in finisher pigs from conventional farms who all used antimicrobials (including tetracyclines) during the finisher stage [114]. In contrast, Græsbøll et al. (2017) found that tetracycline resistance was lower just before slaughter than at earlier time points, although the differences were not statistically significant, and that a significant post weaning peak in resistance was only observed in groups treated with oxytetracycline [115]. These studies provide evidence of the influence of age on AMR, which appears to be independent of AMU. Nevertheless, given the relationship between age and AMU, and the age-related dynamics of the *E. coli* population in the porcine intestinal tract [116,117,118], it is difficult to separate both factors. It is reasonable to attribute this age-related effect to an evolutionary response in the bacterial population, whereby *E. coli* in weaned pigs are adapted to a post weaning intestinal environment which is frequently exposed to antimicrobials. Extended spectrum cephalosporins and fluoroquinolones are notable exceptions to the post weaning peak in resistance, with the higher prevalence observed in piglets [65,119,120] likely influenced by higher exposure to these classes in this age group [107]. Resistance to these classes is discussed separately below.

Relatively few studies have investigated AMR in *E. coli* from sows. Sows represent an important reservoir of AMR on the farm and, although the sow and piglet *E. coli* populations are different [116], associations between resistance in *E. coli* from sows and piglets have been demonstrated in several studies [113,121,122,123,124]. Burow et al. (2019) reported similar levels of resistance in sows and finishers in their study and significant associations between resistance to ampicillin and azithromycin in sows and piglets [113]. In Ireland, the prevalence of AMR in *E. coli* originating from sows was similar to piglets for most of the antimicrobials studied, but higher than in finishers [65]. In contrast, Mathew et al. (2001) reported lower AMR in sows compared with finishers, especially in the ABF herds [114]. Sows on Swiss farms had a lower prevalence of resistance compared with weaners for most of the antimicrobials studied, although not for ciprofloxacin [125]. Two cross-sectional studies in north-eastern Thailand that sampled only sows showed high rates of resistance [126,127] consistent with other studies that sampled finisher pigs in the region [80,84,128]. The NARMS programme includes sampling of sows at slaughter [68] and a recent analysis of the data between 2013 and 2019 showed a generally higher prevalence of AMR in *E. coli* isolated from finisher pigs compared with sows [129]. Such findings contrast with the previously mentioned associations between AMR in sows and their offspring. This apparent contradiction may be influenced by the timing of sampling, i.e., whether sows are lactating or gestating, but studies investigating whether parturition and/or lactation and their associated stresses have an effect on AMR are lacking.

## 5. Mechanisms of Antimicrobial Resistance in *Escherichia coli*

An understanding of the resistance mechanisms employed by *E. coli* is useful in explaining the underlying epidemiology of AMR. Overall, accurate estimates of resistance gene prevalence in the porcine *E. coli* population are difficult to infer from the literature because genotypic studies in the field are carried out less frequently than phenotypic studies, and the methodology may differ in terms of the type of animal sampled and the profile of isolates chosen for evaluation. Furthermore, studies that use PCR methods rely on which gene(s) are chosen for study and thus depend on prior knowledge of the prevailing genotypes. Such prior knowledge is not required for whole genome sequencing (WGS), which allows for an accurate characterisation of the AMR genotype. While studies using WGS to investigate the generic *E coli* commensal population in pigs are still relatively rare, the NARMS programme has performed WGS on a subset of its isolates [68] since 2017, and studies from Europe, Spain and the UK on finisher pigs [130,131,132,133] and from Australia on weaner pigs [134,135] have been published recently. Another study performed a retrospective in silico analysis of *E. coli* WGS data from livestock, including pigs, retrieved from three publicly available genome databases [136]. The latter study reported increasing trends in the prevalence of some ARGs, such as *tet*(A), *bla*_TEM-1b_, *bla*_CMY-2_, and *floR,* over the study period from 1980–2018, although the lack of farm metadata and information on aspects such as sample site or the age of the animal at sampling precludes any farm level analysis of the molecular epidemiology of these resistance mechanisms [136]. The main AMR mechanisms utilised by *E. coli* of animal origin were reviewed by Poirel et al. (2018) [137]. In pigs, tetracycline resistance is most often conferred by the efflux genes *tet*(A) and/or *tet*(B) [68,130,131,132,133,134,135,136], although others such as *tet*(M), a ribosomal protective gene, have been reported [68,81,132,133,138]. Interestingly, Pires et al. (2021) noted the displacement of *tet*(B) by *tet*(A) between 1980 and 2018 in the collection of isolates studied [136], a finding that highlights the dynamic nature of ARG epidemiology. The association of the narrow spectrum beta-lactamase *bla*_TEM-1_ gene (particularly the 1b variant) with ampicillin resistance is an almost universal finding in the studies investigating it [104,130,131,132,133,136,139]. Genes conferring resistance to sulphonamides and trimethoprim, which interfere with bacterial folate metabolism, are also widespread in the porcine *E. coli* population. The prevalence of *sul*1, *sul*2, and *sul*3 varies between studies; *sul*2 was the most common gene in a UK cross sectional study [132] and in the NARMS data [68], *sul*3 was the predominant gene in a Thai study [128], whereas both *sul2* and *sul3* were common on Spanish farms [133]. Streptomycin resistance is associated with the *strA*/*strB* gene pair and with the *aadA* genes (which also confer resistance to spectinomycin); both groups are commonly found on pig farms [130,132,133]. Amphenicol resistance is mainly conferred by the *catA* and *cmlA* genes [140], which persist in the *E. coli* population, despite the fact that chloramphenicol is no longer used in animals in most countries. The *floR* gene is important in the veterinary context as it confers resistance to both florfenicol, which is used in pig farming, and chloramphenicol. Florfenicol resistance is not always reported for studies of commensal isolates, but appears widespread in China [39,141]. In contrast, *floR* was found in 5.5%, 2.9%, and 11.7% of isolates in the UK, USA, and Spain respectively [68,132,133]. The genes conferring resistance to tetracyclines, sulphonamides, trimethoprim, aminoglycosides, and amphenicols represent the most widely distributed ARGs in the *E. coli* population and are frequently co-located. Such co-location explains the phenomenon of multidrug resistance. Indeed, multidrug resistance is a notable feature of many of the studies included in this review and, as an illustrative example, 30.3% of all *E. coli* isolated from pigs at slaughter submitted to the EFSA and ECDC monitoring programme in 2021 were MDR, 44.2% of which were resistant to tetracycline, ampicillin, sulfamethoxazole, and trimethoprim [33]. The relevant ARGs are usually located on mobile genetic elements (MGE) and, in fact, are often co-located on the same MGE. Integrons, especially class 1, have an important role in the epidemiology of MDR [142] as they can capture, express, and exchange ARG cassettes [143] and are frequently located on transposons or plasmids that can facilitate HGT [144]. The most common integron-associated gene cassettes include variants of *aadA* and *dfrA,* but others such *cmlA* are prevalent in pigs [135,145]. Furthermore, the *sul1* gene is part of the conserved 3′ region of the classical class 1 integron structure and *sul3* is also associated with integrons [134,135,146]. This means that isolates harbouring integrons are commonly resistant to sulphonamides, trimethoprim, aminoglycosides, and/or amphenicols. Several studies, especially in Asia, have reported a high prevalence of class 1 integrons of up to 75% in *E. coli* isolates of porcine origin [75,101,105,147,148]. Moreover, although *tet* and *bla*_TEM_ are not found on integrons, they are frequently found in integron positive isolates [147]. 

Despite their abundance (and indeed, because of it), the resistance mechanisms discussed so far are less important in terms of public health, since the associated drugs are no longer routinely used in the treatment of *Enterobacteriaceae* infection of humans. On the other hand, antimicrobials such as third-generation (and higher) cephalosporins, fluoroquinolones, gentamicin, and carbapenems are essential in the treatment of Gram-negative infections and, thus, their respective resistance mechanisms are topics of considerable interest. Gentamicin is a medically important aminoglycoside antibiotic and several genes conferring resistance in *E. coli* have been reported [137]. The *aac*(3)-IVa gene is especially important in the veterinary context as it confers resistance to the veterinary drug apramycin and to gentamicin [137], and its occurrence in human clinical isolates illustrates a rather concrete example of the interface between AMR in animals and humans [149]. The *aac*(3)-IVa gene was the most prevalent gentamicin resistance gene detected in a WGS study carried out on UK finisher herds [132], in two studies in Australia [134,135], and in two older studies in Denmark [150] and Korea [151]. In contrast, the *aadB* gene was common on Thai pig farms [83,128] (the authors did not investigate *aac*(3)-IV), while the NARMS WGS data suggests that *aac*(3)-IId is more prevalent in finisher pigs in the USA [68]. Interestingly, AbuOun et al. (2020) also detected *aac*(3)-IId, but only in fluoroquinolone-resistant isolates recovered from selective media [132]. Tigecycline is a synthetic derivative of tetracycline, developed to treat MDR infections [152]. Resistance, associated with the *tet*(X) ARG, was first reported in isolates of animal origin in China [153], and has been detected in *E. coli* isolates of porcine origin in a number of Chinese studies [80,81] and in five European countries participating in the EFSA and ECDC monitoring programme in 2021 [33]. Carbapenems are of the utmost importance in human medicine as antimicrobial agents of last resort. While still relatively uncommon, resistance genes such as *bla*_VIM_ and *bla*_NDM_ are reported in *E. coli* of porcine origin [33,37,81]. Resistance to the extended spectrum cephalosporins, fluoroquinolones, and polymyxins are discussed separately below.

## 6. Relationship between AMU in Pig Farming and AMR in *Escherichia coli*

### 6.1. Ecological Associations between AMU and AMR in Escherichia coli

Antimicrobial use is generally accepted to be the main driver of AMR and, therefore, the relationship between AMU and AMR in animals is a topic of considerable interest. Ecological studies have identified associations between AMU at a national level and AMR in *E. coli* recovered from national monitoring programmes. Chantziaras et al. (2014) found that the use of specific antimicrobial classes at a national level in seven European countries was correlated with the level of resistance in commensal *E. coli* in cattle, pigs, and poultry [154]. At an individual country level, a trend analysis on data from Belgium between 2011 and 2015 found significant associations between resistance and use of the corresponding class for 10 out the 11 classes studied, while resistance was associated with total AMU for all classes [155]. Both of these studies used species aggregated data and so direct inference for the pig-related data is not possible. However, a Japanese study reported significant correlations between the prevalence of resistance to specific antimicrobial classes in the pig sector and the consumption of the corresponding classes [156], and in Denmark, gentamicin resistance was associated with the consumption of apramycin [152]. More recently, the ‘joint inter-agency reports on integrated analysis of antimicrobial agent consumption and occurrence of antimicrobial resistance in bacteria from humans and food-producing animals in EU/EEA’ (JIACRA) used AMU surveillance data from the European Surveillance of Veterinary Antimicrobial Consumption (ESVAC) project along with AMR data from the EFSA and ECDC monitoring programme to explore the relationship between AMU in livestock and AMR in *E. coli* in Europe [157]. These analyses found significant associations between resistance to fluoroquinolones, colistin, ampicillin, and tetracycline, in indicator *E. coli*, and the use of the corresponding antimicrobial classes at a country level for both the pig specific and the aggregated species datasets [157]. A pan-European study carried out by the EFFORT consortium that sampled 180 farms in nine countries also found significant associations between AMR in *E. coli* and the average treatment incidence at a country level [158]. These associations included cephalosporin use with ampicillin resistance, fluoroquinolone use with ciprofloxacin and nalidixic acid resistance, amphenicol use with chloramphenicol resistance, and lincosamide use with azithromycin resistance [158]. Dorado-Garcia et al. (2016) used species-specific data for a similar analysis in the Netherlands and found that resistance in porcine isolates to a particular antimicrobial was associated with total use rather than use of the corresponding class [159]. Taken together, these studies demonstrate a relationship between AMR in *E. coli* and background AMU at country level. Moreover, they demonstrate that reductions in AMU can lead to reductions in AMR. The Dutch and Belgian studies mentioned previously were conducted during a period of declining AMU in livestock [155,159], and there are similar examples for colistin resistance in China where resistance in *E. coli* and the prevalence of the associated *mcr-1* gene in humans and animals (including pigs) reduced substantially following a ban on the use of colistin as an antimicrobial growth promoter (AGP) [160,161].

### 6.2. Intervention Studies Investigating Relationship between AMU and AMR

Studies investigating the relationship between AMU and AMR can be divided into two categories: intervention studies where the effect of administering an antimicrobial on AMR is investigated, and observational studies where a cohort (or cohorts) of farms are sampled and the results are analysed in conjunction with farm-level AMU data. The former category of study usually takes place on a research farm or on a limited number (often single) of commercial farms, which may allow for a controlled environment, although it may not reflect conditions in the field. Langlois et al. (1978) examined the effect of five in-feed antimicrobial protocols (untreated, bacitracin, virginiamycin, tylosin, and chlortetracycline) on resistance to chlortetracycline (CTC) and found that resistance was lowest in the untreated groups and highest in the groups treated with CTC [162]. In another study, Langlois et al. (1984) found that the response to antimicrobial treatment was affected by the farm’s antimicrobial exposure history [163]. That study evaluated the effect of sub therapeutic and therapeutic doses of CTC on tetracycline resistance in two herds of pigs, one of which was from an ABF farm, and found a greater increase in resistance in the treated groups from the ABF herd compared with the treated groups from the herd with antibiotic exposure [163]. Other studies have demonstrated similar results along with an increase in resistance to unrelated antimicrobials [103,115,164,165]. Delsol et al. (2003) found that tetracycline resistance reduced after treatment was withdrawn, but remained above pre-treatment levels for at least two weeks [164]. Græsbøll et al. (2017) had similar findings, but reported that resistance returned to pre-treatment levels prior to slaughter [115]. A US study investigating resistance to apramycin found that apramycin resistance persisted for longer after apramycin treatment if the pigs had previously been treated with oxytetracycline [166]. Levels of AMR and its persistence were also affected by antimicrobial exposure in the sows [121] and other environmental factors such as temperature, stocking density, and mixing [166,167,168]. These findings are relevant to field studies as there may be a wide variation between farms and, indeed, many of these factors may not be recorded or measurable. Some studies investigated the effect of different treatment regimens in terms of dose and or route of administration. This is of interest because identifying a mode of treatment associated with a lower risk of antimicrobial resistance would be beneficial. Overall, however, the evidence is limited and somewhat contradictory [36]. Increased doses of apramycin were associated with higher aminoglycoside resistance in one study [169]. On the other hand, there was no difference in the response to treatment with different doses of oral or injectable oxytetracycline on five Danish farms [115], which agreed with the conclusions of another Danish study [170]. Similarly, ampicillin resistance in *E. coli* was similar in groups of pigs treated with oral or injectable ampicillin [104]. Interestingly, while the prevalence of phenotypic resistance within *Enterobacteriaceae* did not vary between treatment groups in the latter study, there were higher *Enterobacteriaceae* plate counts and higher gene copy numbers of *bla*_TEM_ detected by qPCR in the faeces of the oral treatment groups [104]. This suggests that mode of treatment affected the *Enterobacteriaceae* and other members of the microbiota differently, and highlights how the quantification of AMR may be subject to different interpretations depending on which methods are used and on the subject population under study. It is also worth noting that this experiment found a rise in multidrug resistance in the *E. coli* population in response to treatment, and that this change was associated with a shift in the phylogenetic profile of the *E. coli* population to phylogroups possessing ampicillin and MDR phenotypes [171]. Interestingly, some studies observed increases in AMR in untreated animals kept in the same pens or rooms as treated animals [115,120,172,173,174]. In one of these studies, fluoroquinolone-resistant *E. coli* strains similar to those found in the fluoroquinolone-treated group were detected in the untreated control group, even though both groups were housed in separate pens [174]. Such findings demonstrate that AMR is influenced by AMU in the community as well as in the individual, and further demonstrate the complexity of AMR epidemiology. Taken together, the intervention studies discussed demonstrate that, in general, antimicrobial treatment causes a rise in AMR in the *E. coli* population and is followed by a decline to pre-treatment levels (in the studies in which this was measured). While these studies provide valuable information on the dynamics of AMR, the findings are not always applicable to the situation in the field, especially as they are usually carried out on a single herd on research farms.

### 6.3. Observational Studies Investigating Relationship between AMU and AMR

Observational studies allow for investigation of the relationship between AMU and AMR at farm level. Ideally, the AMU data used in such studies are as detailed and complete as possible. In practice, such data are not always available and thus categorisation of AMU must be used (e.g., use or not of a particular antimicrobial, high AMU vs. low AMU). Gellin et al. (1989) compared three university farm herds, one that routinely used AGPs, one that only used antimicrobials therapeutically when required, and, lastly, one that did not use any antimicrobials [175]. In almost all cases, resistance to each of the antimicrobials studied was highest on the farm using AGPs and higher on the therapeutic-use farm than the ABF farm [176]. A similar cross-sectional study carried out on seven farrow-to-finish farms, including three ABF farms and four conventional farms in the USA, had similar results [114]. Mathew et al. (1998) also found a higher resistance prevalence on high AMU farms compared with low AMU farms in a longitudinal study conducted from birth to nine weeks of age [176]. Bunner et al. (2007) sampled finisher pigs on 35 ABF and 60 conventional farms in the USA, and reported that the odds of resistance to all antimicrobials were higher on conventional farms, although resistance to quinolones and third generation cephalosporins was absent in both groups [97]. Resistance was also lower on organic farms from four European countries compared with their conventional counterparts [66] and on free range Iberian pig farms compared with conventional farms in Spain [177]. These studies provide evidence for a relationship between AMU and AMR, at least when comparing no or very limited use to higher AMU conventional farms. However, in Southeast Asia, the situation is not as clear cut. In one study in Thailand, resistance to sulfamethoxazole, gentamicin, and chloramphenicol was higher on medium-scale farms (>100 sows) compared with small-scale farms (<100 sows), but resistance to tetracycline was higher on the small-scale farms [127]. A companion study that used the same farms and samples as the previously mention study, but with different laboratory methods, found a higher prevalence of colistin resistance on the small-scale farms [178]. In another, unrelated, Thai study, resistance on farms practising prophylactic AMU was higher than on farms practising only therapeutic use or those with no AMU for most of the antimicrobials investigated, but not for tetracycline or ampicillin [83]. In that study, there were no differences between the therapeutic use farms and ABF farms, and the prevalence of resistance in both groups was, in most cases, higher than the equivalent prevalence on European or North American conventional farms. In fact, the ABF farms were all rural, small-scale farms with no apparent access to veterinary care of any kind [83]. In a similar, but larger-scale study in Thailand, resistance to cephalosporins, azithromycin, and colistin was higher in the prophylactic AMU group compared with those with lower AMU, but the prevalence of resistance to most of the other antimicrobials in all three groups was similar [84]. The high rates of AMR in ABF production systems in these studies could reflect very high background levels of resistance in the region or perhaps reflect unreported or unknown AMU in these herds.

Studies that report the use or not of particular antimicrobial agents or the amounts used allow for a more detailed analysis of the relationship between AMU and AMR. The prevalence of resistance to tetracycline in *E. coli* isolated from pigs of different age groups on Belgian farms was associated with the use of tetracyclines, as well as with the treatment incidence of potentiated sulphonamides [179], which demonstrates both direct and co-selection of resistance associated with AMU. Vieira et al. (2009) also reported a positive association between tetracycline use and tetracycline resistance in Danish pigs at slaughter [180]. Notably, there was a significant association between resistance and the length of time between treatment and slaughter [180], meaning that the closer the last tetracycline treatment was to slaughter, the higher the probability of resistance. Several more studies have reported associations between the use of specific antimicrobials and resistance to their respective classes, albeit not in all instances [65,181,182,183,184,185,186]. These studies also demonstrated associations between the use of specific drugs and resistance to unrelated antimicrobial classes. The use of in-feed antimicrobials in at least one diet was associated with resistance to six out of seven antimicrobials tested on 34 farms in Ontario [181]. The exception was gentamicin resistance, which was only associated with injectable gentamicin use. In four of these models, the association was not antimicrobial specific, meaning resistance increased regardless of which antimicrobial was used, and medicating the starter diet in early weaning was associated with resistance in the finisher stage [181]. The use of medicated feed in grower or finisher diets was also associated with AMR, including MDR, in isolates from finisher pigs in a study in Alberta for four of the investigated models [185]. Similar to the study of Dunlop et al. (1998) [181], there were antimicrobial-specific and non-specific associations with resistance, as well as examples of co-selection [185]. Interestingly, some studies demonstrated associations between macrolide use and unrelated antimicrobial classes, even though *E. coli* is considered intrinsically resistant to macrolides [65,182,185,187]. For example, macrolide use was associated with chloramphenicol resistance on Irish farms [65]. Overall, these studies provide evidence for a complex relationship between AMU and AMR in *E. coli*. Resistance is influenced by AMU both recently and earlier in the lifecycle, as well as by historical usage and background resistance. In many cases, the relationship between AMU and AMR is not clear cut, with some conflicting findings between studies; however, co-selection has an extremely important role. The persistence of chloramphenicol resistance despite a lack of use demonstrates how resistance genes can be maintained in the population due to their association with other resistance genes. An apparent relationship between macrolide use and resistance to other classes further illustrates this complex relationship. The majority of these studies are cross-sectional and, as discussed before, mostly sample pigs at or close to slaughter. A notable exception is the study by Burow et al. (2019), who followed pigs from birth to slaughter on 29 German pig farms [113]. In that study, *E. coli* isolates from pigs treated with penicillins, tetracyclines, polymyxins, or macrolides were more likely to be resistant to ampicillin, tetracycline, colistin, or azithromycin, respectively, than isolates from untreated pigs, but not at all timepoints [113]. In fact, only tetracycline had a significant difference between treated and untreated pigs at the last time point just before slaughter. This shows that studies conducted only in older pigs may not truly capture the relationship between AMU and AMR throughout the whole pig lifecycle.

## 7. Resistance to Extended Spectrum Cephalosporins, Fluoroquinolones, and Polymyxins

Extended spectrum cephalosporins (ESC), fluoroquinolones, and polymyxins (i.e., colistin) are classed as HP CIA by WHO (along with glycopeptides and macrolides) and as ‘Category B’ antimicrobials by the European Medicines Agency (EMA) [9,188]. Although not as heavily used as other antimicrobials and nowadays subject to restrictions in certain settings [189], they are important veterinary antimicrobials, and, in particular, fluoroquinolones and colistin have indications for the treatment of *E. coli* infections in animals. Resistance to these classes in commensal *E. coli* in livestock is especially important because of the importance of these drugs in treating Gram-negative infections in humans and the fact that resistance is well established in both human and animal populations. Isolates resistant to one of these antimicrobials are usually resistant to several other classes of antimicrobial, as illustrated in a study on Belgian poultry and pig farms, where over 90% of ciprofloxacin or cefotaxime-resistant isolates were MDR [190], and in two recent studies that reviewed and conducted meta-analyses of resistance to ESCs, fluoroquinolones, and colistin [37,38]. 

The main mechanisms of ESC resistance are extended spectrum beta lactamases (ESBL) and AmpC beta lactamases, which are, in most cases, associated with MGEs and plasmids [191,192,193]. In humans, ESBLs are the most prevalent and, of these, the CTX-M family is the most important, with the CTX-M-15 and CTX-M-14 variants being the most prominent globally [194,195,196]. Several studies have investigated the prevalence of ESBL/AmpC-producing *E. coli* in pigs, alone or in conjunction with other food producing species. In contrast with humans, there are more distinct geographical patterns regarding the distribution of ESBL/AmpC genes [37]. In East Asia, *bla*_CTX-M-14_, *bla*_CTX-M-55_ and *bla*_CTX-M-65_ predominate [197,198,199,200,201,202,203,204,205,206]. In North America, ESC resistance is usually conferred by the plasmid-mediated AmpC *bla*_CMY-2_ gene, as ESBLs are relatively rare in food-producing animals [37,68,207,208,209]. In Europe, data from the EFSA and ECDC specific monitoring of ESBL/AmpC producing *E. coli* show that the ESBL phenotype is more prevalent than the AmpC phenotype, although this varies between countries [33]. This component of the EFSA and ECDC monitoring programme aims to estimate the prevalence of ESBL/AmpC carriage within the pig population (as opposed to the *E. coli* population) and in 2021, it showed a median prevalence of 54.7%, ranging from 6.5% in Finland to 80.7% in Italy [33]. Comparing these data to the prevalence of ESC resistance in the *E. coli* population (see Table 1) indicates that, although ESBL/AmpC producers are relatively rare within the general *E. coli* population, they are widely distributed within the pig population. Some countries voluntarily submit molecular data to the EFSA and ECDC monitoring programme showing that *bla*_CTX-M-1_ is the most prevalent ESBL gene in Europe [210], which is in agreement with another recent pan European study [211], monitoring programmes in Denmark [32] and the UK [31], and various other European studies [132,212,213,214,215,216]. The EFSA and ECDC data also showed that in the Netherlands and several of the Nordic countries, mutation of the chromosomal *amp*C promoter gene was the predominant ESC-resistance mechanism [210], which perhaps reflects a lower prevalence of plasmid-mediated resistance due to the more restrictive AMU regulations in these countries. This was also the case in Denmark [32]. Ewers et al. (2021) examined 99 ESBL/AmpC-producing *E. coli* isolates recovered from cattle, poultry and pigs at slaughter in eight European countries during the EASSA program [211]. There was marked strain diversity and little evidence of clonality among these isolates, but each ESBL/AmpC gene was associated with particular plasmids, for example, *bla*_CTX-M-1_ with IncI1α, many of which were similar to the sequenced plasmids recovered from *E. coli* in other studies [211]. Although this study included only 15 isolates from pigs (the majority were from poultry), the findings were consistent with several other on-farm studies that showed that ESBL/AmpC genes were distributed across a variety of *E. coli* strains throughout the farm, as shown in the following examples. Moreover, many of these studies demonstrated that on a given farm, usually one ESBL/AmpC gene and plasmid type predominated. Examples include *bla*_TEM-52_ with IncI1, *bla*_CTX-M-1_ with IncN, and *bla*_CTX-M-15_ with IncFIA/FIB on Portuguese farms in two different studies [217,218]; *bla*_CTX-M-1_ with IncN on a Czech farm [219]; *bla*_CTX-M-14_ with IncK2 on a Danish farm [220]; and *bla*_CTX-M-1_ with IncI on an Australian farm [221]. A longitudinal study that followed pigs from birth until slaughter on 31 Swiss farms had broadly similar findings [222]. These studies demonstrate that plasmids carrying ESBL/Amp genes are widely distributed within the *E. coli* population but, at least in Europe, are not yet established in the dominant commensal flora. Other studies have demonstrated a high prevalence of ESC resistance in the general *E. coli* population, for example, one research herd in the USA had a prevalence of resistance to ceftriaxone (associated with *bla*_CMY-2_) between 52.1% and 77.1% in weaner pigs [103], and herds using prophylactic antimicrobials in Thailand had a prevalence of resistance of approximately 35% and 45% in finisher pigs in two separate studies [84,128]. Similarly, several studies from China show a high prevalence of resistance to cephalosporins [75,79,80]. Such studies, even if they are not necessarily applicable to the wider population, show that ESBL/AmpC-bearing plasmids have the potential to establish themselves in the dominant *E. coli* population. As mentioned previously, ESC-resistant *E. coli* are usually multidrug resistant. Most notably, however, in several of the studies discussed in this section, ESC-resistant *E. coli* are frequently co-resistant to fluoroquinolones. This phenomenon is particularly well illustrated in the EFSA and ECDC report: while co-resistance is rare in isolates recovered during routine monitoring (i.e., the general *E. coli* population), 43.3% of the ESC-resistant isolates recovered in specific monitoring of ESBL/AmpC producing *E. coli* during 2021 were resistant to ciprofloxacin [33]. Hayer et al. (2022) examined over 6000 *E. coli* genomes and reported a strong association between *bla*_CTX-M_ and both chromosomal and plasmid-mediated fluoroquinolone-resistant mechanisms [37]. This link between ESC resistance and fluoroquinolone resistance is by no means unique to porcine isolates; it is a notable and concerning problem in human medicine, where, for example, 5.1% of ExPEC isolates submitted to the EARS-Net programme in the EU in 2021 displayed resistance to both classes, along with aminoglycosides [7].

As for studies investigating general antimicrobial resistance in pig production, studies investigating ESC resistance in commensal *E. coli* mainly involve pigs at or close to slaughter. However, several studies have shown that resistance is highest in younger pigs. There was a higher prevalence of ESC-resistant *E. coli* carriage in piglets compared with older age groups in four longitudinal studies [119,123,124,223]. Bacterial counts of ESC-resistant *E. coli* also decreased with age [119,124,223,224,225], although this measurement did not consider the proportion of the population it represents. Other studies in Asia showed a higher prevalence of resistance in weaner pigs [128,226]. Resistance to ESCs has been associated with AMU in some studies. While cephalosporin use at a national level in Europe was not associated with the prevalence of cefotaxime or ceftazidime resistance in the general *E. coli* population, it was associated with the prevalence of ESBL/AmpC carriage [157]. At farm level, resistance was more likely on farms with cephalosporin use compared with those without use [227,228,229]. Agersø and Aarestrup reported a significant reduction in the prevalence of ESC resistance in Danish slaughter pigs after a voluntary ban on cephalosporin use within the industry [230]. There were similar findings over a four year period on an Australian farm that had ceased cephalosporin use [221]. 

In comparison with ESC resistance, there are fewer studies investigating fluoroquinolone resistance in the porcine commensal *E. coli* population. Fluoroquinolones (e.g., ciprofloxacin) have been included in the AST panels in most studies; however, different interpretation criteria and changes in breakpoints over time hamper comparability somewhat. In particular, whether ECOFFs or CBPs are used greatly influences this interpretation as the EUCAST-defined ciprofloxacin cut off MIC for wild type *E. coli* is currently 0.06 mg/L [42] compared with a CBP (for resistant) of >0.5 mg/L [40,43]. Therefore, studies using ECOFFs typically report a higher prevalence of fluoroquinolone resistance than those using higher breakpoints, as can be seen in the EFSA and ECDC data presented in Table 1. The targets for (fluoro)quinolones are DNA gyrase and topoisomerase IV, which are involved in DNA replication, transcription, and repair. Target modification caused by mutations in the quinolone-resistance determining region (QRDR), usually of *gyr*A and/or *par*C, can confer resistance to quinolones, but only low-level resistance to fluoroquinolones [231]. These can be detected if using ECOFFs. Further mutations are required for clinical resistance. Plasmid-mediated quinolone resistance (PMQR) mechanisms such as the *qnr* family, which protects DNA gyrase and topoisomerase, or genes encoding efflux pumps such as *qepA* or *oxq*AB, by themselves confer a low level fluoroquinolone resistance, but facilitate mutations in the QRDR by ‘lengthening’ the mutation prevention window [231,232]. In pigs, chromosomal mutations of *gyr*A, followed by *par*C, are the most frequently encountered fluoroquinolone resistance mechanisms while *qnrS1* and *qnrB19* are the most commonly encountered plasmid-mediated mechanisms [38,68,132,133]. The rates of fluoroquinolone resistance in finisher pigs are typically lower than the rates for the ‘older’ antimicrobials and often higher than ESC resistance (see Table 1 and Table 2). As for ESC resistance, fluoroquinolone resistance is widely distributed in the pig population. In the UK, for example, one cross sectional study found a high level ciprofloxacin resistance on 58% of finisher farms [233], and a more recent WGS-based study found QRDR mutations on 78.5% of farms [132]. Studies investigating fluoroquinolone resistance in younger pigs are scarce. The highest prevalence of fluoroquinolone resistance was found in piglets in an Irish cross-sectional study [65], but was highest in weaner pigs in two smaller-scale longitudinal studies in Vietnam and Brazil [110,226]. Recently, a larger-scale longitudinal study on 24 Swiss farms investigated nalidixic acid resistance in fluoroquinolone-treated and untreated pigs, and found the highest number of positive samples in piglets [120]. Resistance prevalence was also higher in the treated groups and lower on farms not using any fluoroquinolones. Associations between fluoroquinolone use and resistance have also been demonstrated at national- and international level [157,158]. 

Colistin resistance has become a topic of increasing interest recently. Previously reserved for animal use due to nephrotoxicity in humans, it is now an agent of last resort used to treat some carbapenem-resistant infections in people [234]. Resistance to colistin was previously thought to be chromosomally mediated until the discovery of the plasmid-mediated *mcr* gene in porcine isolates in China [235]. To date, 10 variants of the *mcr* gene have been described [236], although the original *mcr-1* variant predominates worldwide [38]. While numerous studies have investigated colistin resistance in *E. coli* of porcine origin [38], only a few have explored the on-farm dynamics. One longitudinal study in Vietnam reported a lower prevalence of colistin resistance in piglets compared with weaners or finishers [226], while a cross-sectional study in Korea showed a higher prevalence of resistance in weaners compared with the older age groups [237]. As seen in Table 1, colistin resistance in *E. coli* isolated from finishers in the developed world is currently rare; however, as previously discussed, many studies in Asia show a higher prevalence of resistance. In Europe, the prevalence of colistin resistance in pigs at slaughter is associated with the use of colistin at a national level [157]. 

## 8. Conclusions and Future Perspectives

In summary, while there is a considerable body of research characterising antimicrobial resistance in porcine *E. coli*, its relationship with AMU and, to a lesser extent, its evolution during the production cycle, knowledge gaps remain. In particular, longitudinal studies are lacking, especially for fluoroquinolone and colistin resistance. Although the point of slaughter is considered most relevant to human health, AMR in younger pigs, in which resistance is typically higher, is extremely important as this influences AMR at finishing, and is relevant to human occupational exposure and environmental contamination. Thus, more on-farm cross-sectional studies that sample younger pigs or longitudinal studies encompassing the full lifecycle are warranted. The majority of the studies included in this review were conducted in Europe, North America, and, to a lesser extent, China and Southeast Asia. This highlights a lack of data from LMICs and future studies from such countries or regions would be beneficial. In particular, the implementation of integrated monitoring programmes in developing countries is a pressing need as AMU in livestock production is expected to increase in these countries as their food production sectors expand [22]. However, strengthening the surveillance and research of AMR in agriculture is a key component of the Food and Agriculture Organization’s (FAO) action plan on AMR [238], and the successful implementation of such programmes in LMICs will provide essential data to improve the understanding of the epidemiology of AMR in *E. coli* and other bacteria globally. There is also a need for an increased understanding of the molecular epidemiology of AMR in the general commensal *E. coli* population as genomic studies, unsurprisingly, tend to focus on the ARGs most important to human health such as ESBL/AmpC, carbapenemases, and mobile resistance genes to fluoroquinolones (e.g., *qnr*) and polymyxins (i.e., *mcr*-1). However, the EFSA and ECDC monitoring programme now provides for the use of WGS data [33], and the increasing availability and affordability of WGS is likely to provide intriguing new insights on this topic.

This review was motivated by the potential role played by AMR commensal *E. coli* of animal origin in the overall epidemiology of AMR. While its overall importance in the overall context of AMR remains to be established, one key question for the future is how will AMR in *E. coli* evolve in the coming years? On the one hand, several European countries show decreasing trends in resistance to some antimicrobials as their efforts to increase awareness of AMR and improve antimicrobial stewardship take effect [33]. On the other hand, AMU in livestock is projected by some authors to increase in line with the increasing global demand for food [22], and LMICs in Asia have already been identified as ‘hotspots’ for AMR in livestock [37,38,61]. Ultimately, whether this potential threat to human health can be mitigated or reduced depends on the engagement and concerted action of all stakeholders within the health and agriculture sectors. Continued research, monitoring and surveillance will be required into the future to underpin these efforts. 

## Figures and Tables

**Table 1:** Summary of antimicrobial resistance in commensal *Escherichia coli* of porcine origin extracted from the most recent data of monitoring programmes in the EU/EEA, UK, USA, Canada, and Japan. Data from selected European countries participating in the EFSA and ECDC monitoring programme are included.

		Antimicrobial ^a^															
Country	Year ^b^	TET	SUL ^c^	TMP	SXT	AMP	CHL	STR	GEN	AXO ^d^	CTX ^d^	CIP ^d^	CIP HL ^d^	AZM ^d^	COL ^d^	CS	MDR
Denmark	2021	29.2%	41.5%	30.8%	-	38.5%	4.6%	-	0.0%	-	1.5%	0.0%	-	4.6%	0.0%	52.3%	33.8%
France	2021	42.2%	29.3%	21.1%	-	25.4%	8.6%	-	0.0%	-	0.9%	2.6%	0.4%	1.3%	0.0%	44.0%	23.7%
Germany	2021	32.1%	29.5%	23.7%	-	28.9%	6.3%	-	2.6%	-	0.0%	1.6%	0.5%	2.6%	0.0%	49.5%	22.6%
Ireland	2021	51.8%	37.1%	36.5%	-	28.2%	9.4%	-	3.5%	-	0.0%	2.4%	0.0%	0.6%	0.0%	38.2%	32.9%
Netherlands	2021	31.0%	24.0%	24.3%	-	22.3%	9.3%	-	0.7%	-	0.0%	2.0%	0.0%	1.7%	0.0%	50.7%	20.3%
Spain	2021	78.8%	58.8%	60.0%	-	83.5%	41.2%	-	4.7%	-	1.2%	50.6%	11.8%	4.7%	0.0%	6.5%	78.8%
Sweden	2021	16.8%	22.5%	19.7%	-	24.9%	7.5%	-	0.0%	-	0.6%	1.7%	0.0%	0.6%	0.0%	63.6%	19.7%
**EU/EEA ^e^**	**2021**	**45.9%**	**33.8%**	**25.9%**	**-**	**32.8%**	**11.8%**	**-**	**1.1%**	**-**	**0.9%**	**6.4%**	**1.2%**	**1.6%**	**0.0%**	**38.3%**	**31.2%**
UK ^f^	2021	52.7%	40.5%	37.6%	-	33.3%	18.6%	-	2.1%	-	1.3%	4.6%	-	-	0.0%	-	-
USA ^g^	2021	66.5%	20.3%	-	9.7%	25.0%	7.2%	-	3.8%	9.3%	-	10.2%	3.0%	0.4%	-	27.1%	16.1%
Canada ^h^	2019	55.5%	35.1%	-	13.1%	29.9%	12.4%	40.9%	0.0%	2.2%	-	-	0.0%	0.0%	-	25.5%	-
Japan ^i^	2017	55.4%	-	-	26.5%	33.7%	21.7%	41.0%	3.6%	-	1.2%	-	-	-	0.0%	-	-

^a^ Antimicrobials: TET—tetracycline; SUL—sulfamethoxazole or sulfisoxazole (see ^c^); TMP—trimethoprim; SXT—trimethoprim/sulfamethoxazole; AMP—ampicillin; CHL—chloramphenicol; STR—streptomycin; GEN—gentamicin; AXO—ceftriaxone; CTX—cefotaxime; CIP—ciprofloxacin (MIC > 0.06 mg/L); CIP HL—ciprofloxacin (MIC > 1 mg/L); AZM—azithromycin; COL—colistin; CS—complete susceptibility to all antimicrobials tested; MDR—multidrug resistance, resistance to antimicrobials in three or more classes. ^b^ Year of sampling. ^c^ Sulfamethoxazole is the sulphonamide representative in the EFSA and ECDC testing panel. Sulfisoxazole is the sulphonamide representative in the NARMS and CIPARS panels. ^d^ Highest Priority Critically Important Antimicrobial [8] ^e^ Participating EU/EEA countries submit data every two years to the EFSA and ECDC monitoring programme. Data from seven selected countries of interest are shown. The overall EU/EEA data, highlighted in bold, represents the median for all 32 participating countries. Antimicrobial susceptibility interpreted according to ECOFFs defined by EUCAST [33]. ^f^ United Kingdom Veterinary Antimicrobial Resistance and Sales Surveillance 2021 (UK-VARSS). Antimicrobial susceptibility interpreted according to ECOFFs defined by EUCAST [31]. ^g^ The National Antimicrobial Resistance Monitoring System (NARMS). Antimicrobial susceptibility interpreted according to CLSI M100-Ed30 [68]. ^h^ Canadian Integrated Program for Antimicrobial Resistance Surveillance (CIPARS). Antimicrobial susceptibility interpreted according to CLSI M100-S26 [87]. ^i^ Japanese Veterinary Antimicrobial Resistance Monitoring System (JVARM). Antimicrobial susceptibility interpreted according to CLSI M100-S27 [88]. Legend: CLSI—Clinical and Laboratories Standards Institute; ECOFF—Epidemiologic cut off value; EEA—European Economic Area; EU—European Union; EUCAST—European Committee on Antimicrobial Susceptibility Testing; UK—United Kingdom; USA—United States of America.

**Table 2:** Summary of resistance to selected antimicrobials in commensal *Escherichia coli* from healthy pigs at or before slaughter, extracted from selected published studies.

Study		Antimicrobial ^a^											
Country	Year ^b^	TET	SUL	TMP	SXT	AMP	STR	GEN	CHL	CTX	TIO	AXO	CIP
*Europe*													
Spain [89]	2000	95.6%	87.8%	83.4%	-	72.2%	-	8.0%	59.5%	-	-	-	-
Spain [90]	2001	68.0%	-	-	48.0%	29%	-	7.0%	15.0%	0%	-	-	3.0%
Portugal [63]	2013	93.9%	-	-	69.7%	68.2%	77.3%	4.5%	36.4%	0%	-	-	1.5%
Poland [64]	2013	48.9%	35.8%	16.3%	-	29.5%	42.6%	2.6%	18.9%	2.6%	-	-	6.3%
Ireland [65]	2016	59.0%	-	-	27.6%	18.0%	33.3%	5.8%	9.6%	0%	0%	-	0%
Estonia [67]	2019	32.5%	30.0%	22.4%	-	58.7%	39.2%	12.5%	5.8%	2.5%	-	0%	5.8%
Denmark ^c^ [66]	2016	42.3%	24.6%	23.1%	-	25.0%	44.2%	5.8%	0%	0%	-	-	0%
France ^c^ [66]	2016	74.5%	-	-	40.4%	14.9%	66.0%	7.5%	17.0%	0%	-	-	4.3%
Italy ^c^ [66]	2016	74.4%	61.6%	50.4%	-	62.4%	61.6%	6.4%	30.4%	0%	-	-	12.0%
Sweden ^c^ [66]	2016	14.1%	25.4%	19.7%	-	18.3%	25.4%	1.4%	1.4%	0%	-	-	1.4%
Denmark [91]	2012	36.0%	-	-	14.7%	24.0%	-	0%	6.7%	0%	-	-	0%
France [91]	2012	83.2%	-	-	43.6%	24.8%	-	2.0%	20.8%	0%	-	-	0%
Germany [91]	2012	64.4%	-	-	33.7%	33.7%	-	0%	13.5%	0%	-	-	1.0%
Netherlands [91]	2012	67.9%	-	-	42.1%	25.7%	-	0%	14.3%	0%	-	-	0%
Spain [91]	2012	94.0%	-	-	66.0%	66.0%	-	5.0%	42.0%	0%	-	-	0%
Europe ^d^ [92]	2022	53.3%	-	-	29.5%	35.3%	-	2.2%	21.3%	0.8%	-	-	1.6%
France ^d^ [92]	2022	61.7%	-	-	29.0%	29.4%	-	0.5%	13.6%	0.5%	-	-	0.9%
Germany ^d^ [92]	2022	32.9%	-	-	18.6%	27.1%	-	0.5%	6.2%	2.9%	-	-	1.0%
Netherlands ^d^ [92]	2022	43.1%	-	-	24.1%	24.5%	-	0.5%	31.0%	0.0%	-	-	0.5%
Spain ^d^ [92]	2022	81.6%	-	-	50.2%	66.7%	-	5.5%	39.8%	0.5%	-	-	6.0%
UK ^d^ [92]	2022	48.5%	-	-	26.5%	30.4%	-	4.4%	16.7%	0.0%	-	-	0.0%
*North America*													
Canada [93]	1998	71.3%	38.2%	-	-	29.1%	-	0.6%	-	-	-	-	-
Canada [94]	2008	78.9%	49.9%	-	6.4%	30.6%	49.6%	1.1%	17.6%	-	0%	0%	0%
Canada [95]	2008	66.8%	46.0%	-	7.4%	18.6%	33.4%	0.8%	17.3%	-	0.1%	0%	0%
Canada [96]	2008	73.0%	46.6%	-	2.6%	25.7%	27.5%	0.4%	15.5%	-	0%	0.43%	0%
USA ^c^ [97]	2007	90.9%	31.6%	-	1.9%	24.1%	28.9%	0.8%	8.2%	-	0.3%	2.0%	0%
*Australia*													
Australia [98]	2016	-	-	-	-	29.4%	-	17.5%	-	-	1.8%	-	-
Australia [99]	2018	67.7%	-	-	34.3%	60.2%	33.9%	0%	22.4%	-	0%	0%	1.0%
*Asia*													
Korea [100]	2007	96.3%	-	-	38.8%	66.1%	66.8%	42.0%	47.6%	1.0%	-	-	7.8%
Korea ^d^ [101]	2014	89.9%	-	-	-	71.3%	61.2%	23.2%	68.4%	-	-	-	8.4%
Korea ^d^ [102]	2022	73.9%	-	-	84.8%	79.4%	74.5%	17.6%	80.0%	-	5.5%	-	14.5%
China [74]	2019	98.3%	-	-	71.6%	90.0%	-	21.7%	75.0%	-	-	-	21.7%
China [75]	2020	73.5%	-	-	71.6%	58.0%	53.0%	21.6%	-	16.7%	-	-	23.9%
*Africa*													
Tanzania [72]	2015	72.9%	-	-	60.0%	38.6%	50.0%	-	-	24.3%	-	-	10%
Tanzania [73]	2021	51.3%	-	-	47.7%	46.4%	-	26.0%	27.3%	29.5%	-	-	28.6%
Nigeria [69]	2015	50.0%	-	-	17.9%	10.7%	-	0%	-	3.6%	-	3.6%	3.6%
Rwanda [71]	2021	26.7%	-	-	-	12.6%	13.3%	-	2.2%	0.7%	-	0.7%	0%
Uganda [70]	2021	53.9%	88.5%	17.3%	-	11.5%	-	3.8%	5.7%	7.7%	-	-	7.6%

^a^ Antimicrobial: TET—tetracycline; SUL—sulfamethoxazole or sulfisoxazole; TMP—trimethoprim; SXT—trimethoprim/sulfamethoxazole; AMP -ampicillin; STR—streptomycin; GEN—gentamicin; CHL—chloramphenicol; AXO—ceftriaxone; TIO—ceftiofur; CTX—cefotaxime; CIP—ciprofloxacin, ^b^ Year of publication ^c^ Study compared conventional and organic/antibiotic free systems. Results from conventional farms only are shown, ^d^ Multi-year study: results from final year of study are shown. Legend: UK—United Kingdom; USA—United States of America.

## Data Availability

Data are contained within the article.

## Abbreviations

ABF—antibiotic free; AGISAR—Advisory Group on Integrated Surveillance of Antimicrobial Resistance; AGP—antimicrobial growth promoter; AMR—antimicrobial resistance; AMU—antimicrobial use; APEC—avian pathogenic *Escherichia coli*; ARG—antimicrobial resistance gene; AST—antimicrobial susceptibility testing; CBP—clinical breakpoint; CIA—critically important antimicrobial; CIPARS—Canadian Integrated Program for Antimicrobial Resistance Surveillance; CLSI—Clinical and Laboratories Standards Institute; CTC—chlortetracycline; DANMAP—Danish Integrated Antimicrobial Resistance Monitoring and Research Programme; DNA—deoxyribonucleic acid; EARS-Net—European Antimicrobial Resistance Surveillance Network; EASSA—European Antimicrobial Susceptibility Surveillance in Animals; ECDC—European Centre for Disease Prevention and Control; ECOFF—epidemiologic cut off value; EEA—European Economic Area; EFFORT—Ecology from Farm to Fork Of microbial drug Resistance and Transmission; EFSA—European Food Safety Agency; EMA—European Medicines Agency; ESBL—extended spectrum beta lactamase; ESC—extended spectrum cephalosporin; ESVAC—European Surveillance of Veterinary Antimicrobial Consumption; ETEC—enterotoxigenic *Escherichia coli*; EU—European Union; EUCAST—European Committee on Antimicrobial Susceptibility Testing; ExPEC—extra intestinal pathogenic *Escherichia coli*; FAO—Food and Agriculture Organization; FDA—Food and Drug Administration; HGT—horizontal gene transfer; HP CIA—highest priority critically important antimicrobial; JIACRA—joint inter-agency reports on integrated analysis of antimicrobial agent consumption and occurrence of antimicrobial resistance; JVARM—Japanese Veterinary Antimicrobial Resistance Monitoring System; LMIC—lower and middle income countries; MARAN—Monitoring of Antimicrobial Resistance and Antibiotic Usage in Animals in the Netherlands; MDR—multidrug resistant/multidrug resistance; MGE—mobile genetic element; MIC—minimum inhibitory concentration; NARMS—National Antimicrobial Resistance Monitoring System; PCR—polymerase chain reaction; PMQR—plasmid-mediated fluoroquinolone resistance; QRDR—quinolone resistance determining region; STEC—Shiga toxin producing *Escherichia coli*; UK—United Kingdom; UK-VARSS—United Kingdom Veterinary Antimicrobial Resistance and Sale Surveillance; USA—United States of America; WGS—whole genome sequencing; WHO—World Health Organization; WT/nWT—wild type/non-wild type.
